# Knowledge and Perceptions of AI Among Medical Students in Morocco: Cross-Sectional Study

**DOI:** 10.2196/66156

**Published:** 2025-09-19

**Authors:** Imad Chakri, Otmane El Khayali, Laila Lahlou

**Affiliations:** 1 Faculty of Medicine and Pharmacy Agadir, University Ibn Zohr, Quartier Tilila Bp 7519 Agence Abb Agadir Al Fidia, Agadir, 80060, Morocco, 212 528227170, 212 528217171

**Keywords:** knowledge, perception, medical education, medical knowledge, medical training, medical students, machine learning, ML, artificial intelligence, AI, large language models, LLM, natural language processing, NLP, deep learning, cross-sectional studies, observational studies, Morocco

## Abstract

**Background:**

Artificial intelligence (AI) is rapidly transforming medical practice by enhancing diagnostic accuracy, streamlining workflows, and supporting clinical decision-making. However, the integration of AI into health care largely depends on the preparedness and acceptance of future physicians. Therefore, assessing their knowledge and perceptions of AI is crucial. Notably, no study has yet evaluated these factors among medical students in Morocco.

**Objective:**

The aim of this study was to describe Moroccan medical students’ knowledge and perception of AI.

**Methods:**

A cross-sectional, observational study was conducted from February to May 2023 at the Faculty of Medicine and Pharmacy, Agadir, Morocco. All undergraduate medical students from the first to the seventh year were eligible, excluding graduate students. A snowball sampling method was used, with a calculated minimum sample size of 385. To account for potential missing data, and given the target population size of 1150, the sample size was increased by 50%. Data were collected through a validated online questionnaire and analyzed using JAMOVI 2.6.2, with significance set at *P*<.05.

**Results:**

A total of 580 medical students (female n=363, 62.6%; mean age 21.3, SD 2.13 years; response rate 50.4%) participated. While 96% (n=557) had heard of AI, 73.1% (n=424) were unfamiliar with key AI terminologies, only 11% (n=64) understood AI functioning, and 14.8% (n=86) were familiar with everyday AI applications. Objectively, 88.1% (n=511) correctly identified deep learning as a method for automated pattern recognition, with 71.5% (n=415) acknowledging its interpretability challenges. First-cycle students demonstrated significantly higher familiarity with AI terms (83/156, 53.2% vs 51/156, 32.7% vs 22/156, 14.1%; *P*<.001). In terms of perception, 83% (n=482) viewed AI as a collaborative tool, 84.1% (n=488) anticipated a transformative impact on medicine, 39% (n=227) expected noninterventional medicine to be replaced within a decade, and 57.1% (n=331) believed certain specialties could be supplanted by AI. Regarding AI in medical education, 90% (n=522) supported its integration into the curriculum and 94% (n=546) expected enhanced learning conditions, but only 48.1% (n=279) felt ready to use AI tools upon graduation. Additionally, gender and technology familiarity significantly influenced specific perceptions, with technology-savvy students reporting greater readiness (*P*<.001) and women more likely to view AI as revolutionary (315/488, 64.5% vs 173/488, 35.5%; *P*=.02).

**Conclusions:**

Medical students’ knowledge of AI is still limited, but their awareness of the potential impact of this technology on future practice and their openness to its integration into the medical curriculum constitute a promising basis for the successful implementation of these new concepts in our health care system.

## Introduction

Artificial intelligence (AI) is commonly defined as the capability of machines to perform tasks that typically require human intelligence, such as reasoning, learning, problem-solving, and decision-making [1]. AI encompasses various subfields, including machine learning (ML), which enables systems to identify patterns and improve performance through experience without explicit programming, and deep learning (DL), a specialized branch of ML that utilizes artificial neural networks to analyze complex data structures with high accuracy [2].

Recent advancements in computational power, big data analytics, and algorithmic efficiency have accelerated AI’s integration across multiple domains, including finance, education, and transportation [3-5]. In health care, AI has demonstrated remarkable potential in diagnostics, predictive modeling, medical imaging, and personalized treatment, paving the way for a paradigm shift in clinical practice and medical education [6-8].

However, despite its transformative potential, AI also introduces critical challenges related to ethics, data security, and regulatory compliance, necessitating careful oversight to ensure responsible and equitable implementation in health care settings. Concerns regarding patient data privacy, algorithmic bias, and the interpretability of AI-driven decisions highlight the need for stringent regulatory frameworks and ethical guidelines to govern AI applications in medicine [9-12]. Additionally, AI adoption in health care requires well-trained professionals who can critically evaluate and effectively utilize AI-driven tools while ensuring patient safety and maintaining ethical standards.

Given the increasing role of AI in modern health care, medical education must evolve to equip future physicians with the necessary knowledge and skills to integrate AI into clinical practice. Various international organizations, including the World Medical Association, have advocated for incorporating AI-related topics into medical curricula to enhance AI literacy among health care professionals [13]. Despite these recommendations, AI education remains largely absent from undergraduate medical training in many countries, including Morocco, leaving medical students with limited exposure to AI concepts, applications, and ethical implications.

This study aimed to describe the knowledge and perceptions of medical students in Morocco regarding AI. It seeks to assess their familiarity with AI applications in medicine, their perceptions of AI’s role in medical practice, and the potential impact of AI on their career choices.

## Methods

### Design

A cross-sectional study was conducted among undergraduate medical students at the Faculty of Medicine and Pharmacy, Agadir (FMPA), Morocco, from February 1, 2023, to May 1, 2023. An online self-administered questionnaire served as the principal instrument for data collection. All undergraduate students were included. Graduate students were excluded because, at the time of the study, FMPA had not yet had any graduates. Students from other medical faculties were also excluded. There were no exclusion criteria based on age or gender.

### Sampling

A snowball sampling method was employed. The minimum sample size was calculated with a 5% margin of error, 5% precision, 95% confidence level [14,15], and an estimated AI knowledge prevalence of 50% among medical students. This estimate is based on a study in Saudi Arabia [16], which found that approximately 50% of students believed they had a good understanding of AI; however, when knowledge of AI was tested, only 22% of the questions were answered correctly. The required sample size was determined to be at least 385 students. To account for potential missing data, and given that the target population size was 1150, we increased the sample size by 50%.

### Questionnaire Development and Validation

The questionnaire was created using commonly used questions from previous studies [17-19] and reviewed by the authors to ensure clarity, completeness, and relevance, with specific improvements made based on feedback. To validate the clarity of the questions, a small pilot group of 10 students completed the survey.

The survey is structured into 32 questions across 5 sections:

- Demographics (4 questions): This section collects essential demographic information, including age, gender, educational level, and study location.
- Knowledge of AI (12 questions): Participants are asked about their sources of information on AI, familiarity with AI terminology, understanding of AI applications in daily life, awareness of AI investments in health care, and interest in AI among medical professionals.
- Perceptions of AI (7 questions): This section explores participants’ views on whether AI will collaborate with or compete against health care providers, its potential impact on medicine, concerns about AI replacing doctors, and overall attitudes toward AI development.
- AI in medical education (6 questions): Participants are asked about the perceived benefits of AI in medical education, potential changes in teaching methods due to AI, and related considerations.
- Influence of AI on specialty choice (3 questions): This section examines participants’ opinions on which medical specialties will be most affected by AI, concerns about AI replacing doctors in certain specialties, and whether AI advancements might influence their choice of medical specialty.

### Data Collection

Data were collected using a self-administered online questionnaire developed on the Google Forms platform and written in French. The questionnaire employed straightforward and comprehensible language, disseminated through social media channels, particularly on WhatsApp groups comprising students from the first to the seventh year at FMPA.

### Ethical Considerations

#### Ethics Approval and Review

This study was conducted in accordance with the ethical principles outlined in the Declaration of Helsinki (1964) and its subsequent amendments [20]. As there is currently no institutional review board or ethics committee at our faculty, the study was conducted following institutional ethical guidelines and best practices for research involving human participants.

#### Informed Consent

Participation in this study was entirely voluntary. All participants were informed about the study’s objectives, methods, and their rights, including the right to withdraw at any time without any consequences. Informed consent was obtained from each participant before data collection. Since the study involved secondary data analysis, consent for the original data collection was obtained, and its use for this research was conducted in line with ethical guidelines.

#### Privacy and Confidentiality

The study ensured complete anonymity and confidentiality of participants. No identifiable personal data were collected, and all information was deidentified prior to analysis. Data were securely stored and accessible only to authorized research personnel to prevent unauthorized access.

#### Compensation

Participants did not receive any financial or material compensation for their participation in this study.

#### Participant Identifiability in Images or Supplementary Materials

This study does not include any images or supplementary materials that could lead to the identification of individual participants. If any identifiable images were to be included, explicit consent would be obtained, and the relevant consent forms would be submitted as supplementary material.

### Data Analysis

Data analysis was conducted using JAMOVI (version 2.6.2) [21,22]. Descriptive statistics summarized qualitative variables in terms of frequencies and percentages, while the quantitative variable (age) was described using mean (SD). Comparative analyses were performed using the Student *t* test for independent samples when comparing two groups and 1-way ANOVA when comparing 3 or more groups. The choice between the *t* test and ANOVA was based on the number of groups being compared. For categorical variables, the *χ*^2^ test or Fisher exact test was applied depending on the expected frequency of the data. This allowed assessment of students’ knowledge of AI in medical education, the influence of AI on specialty choice, and predictors of perceptions (including demographics such as gender, grade, and technology experience). A *P* value <.05 was considered statistically significant.

## Results

### Demographic Characteristics

**Figure 1.**
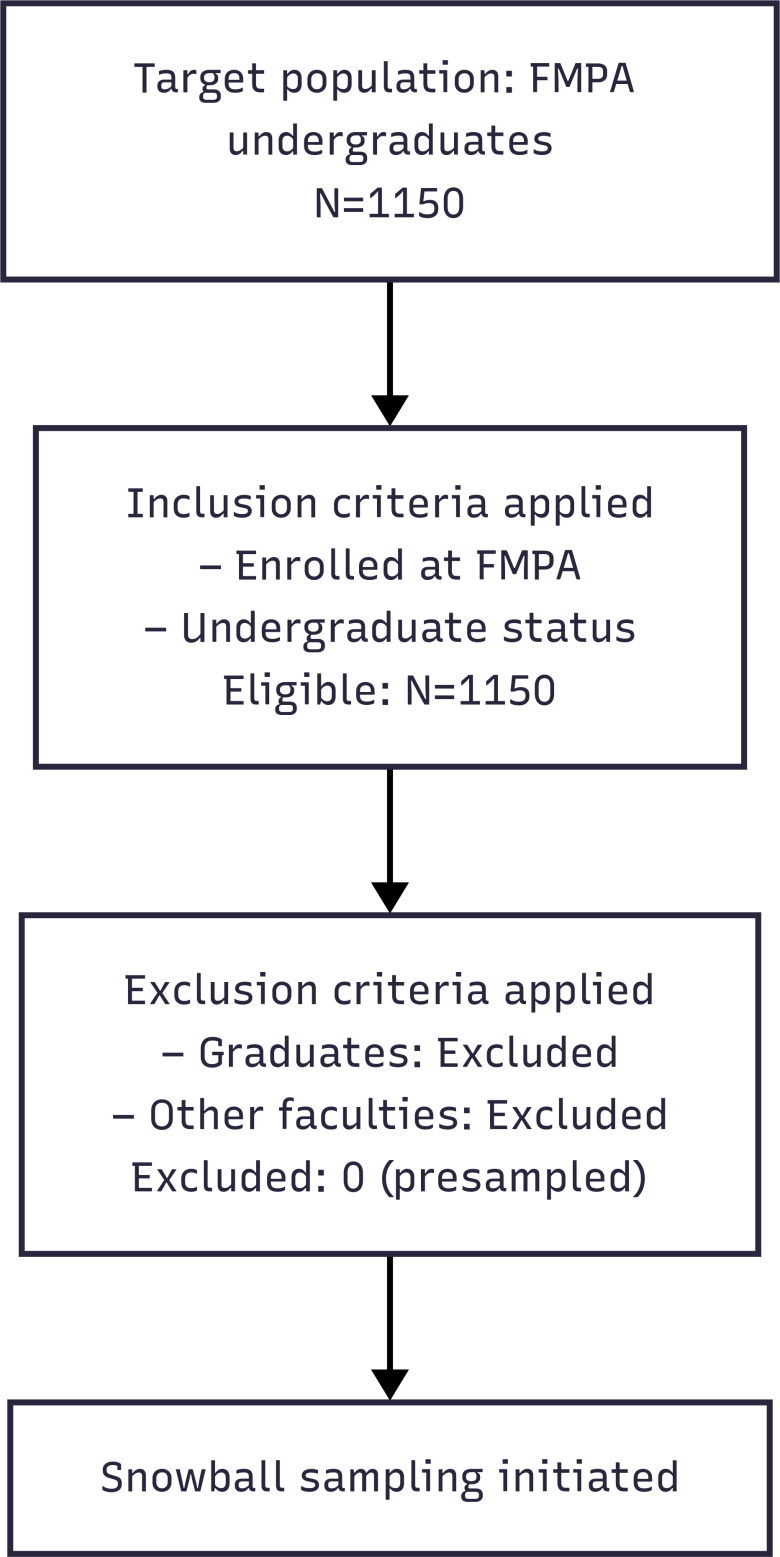
Flowchart of participant inclusion and exclusion criteria. FMPA: Faculty of Medicine and Pharmacy, Agadir.

Figure 1 illustrates the participant flow, including the inclusion and exclusion criteria applied during the sampling process. A total of 580 medical students participated in our survey, yielding a response rate of 50.4%. Most of the participants were female, comprising 363 (62.6%) individuals. The mean age of the participants was 21.3 (SD 2.13) years, with an age range from 18 to 26 years.

Participants were distributed across various academic years as follows: 19.1% (n=111) were first-year students, 17.6% (n=102) second-year, 13.8% (n=80) third-year, 13.8% (n=80) fourth-year, 11.7% (n=68) fifth-year, 11.9% (n=69) sixth-year, and 12.1% (n=70) seventh-year students. Regarding academic cycles, 36.7% (n=213) of students were enrolled in the first cycle (first and second years), 39.3% (n=228) of students in the second cycle (third, fourth, and fifth years), and 24% (n=139) of students in the third cycle (sixth and seventh years). The demographics of the included participants are shown in Table 1.

**Table 1.** Demographic characteristics of the medical students (n=580).

Characteristics	Values
Gender, n (%)	
Female	363 (63.6)
Male	217 (37.4)
Age (years)	
Mean (SD)	21.3 (2.13)
Range	18‐26
Academic year, n (%)	
First year	111 (19.1)
Second year	102 (17.6)
Third year	80 (13.8)
Fourth year	80 (13.8)
Fifth year	68 (11.7)
Sixth year	69 (11.9)
Seventh year	70 (12.1)
Academic cycle, n (%)	
First cycle (first and second years)	213 (36.7)
Second cycle (third, fourth, and fifth years)	228 (39.3)
Third cycle (sixth and seventh years)	139 (24.0)

### Knowledge of AI

Regarding knowledge, 249 (42.9%) of the 580 participating medical students declared themselves knowledgeable about technology. Almost all students (n=557, 96%) had heard of AI, with social media being the primary source for 545 students, followed by traditional media such as television, radio, and journals for 350 students; scientific journals for 236 students; and universities for 150 students. However, a notable 73% (n=423) of participants were unfamiliar with basic AI terminology such as machine learning, deep learning, and neural networks. Only 11% (n=64) of students claimed to have an understanding of how AI functions. Additionally, a mere 14.8% (n=86) reported familiarity with everyday AI applications like speech recognition and antispam filters.

The association of knowledge of AI with different variables is given in Table 2. Female participants showed higher self-perceived familiarity with technology (131/248, 52.8% vs 117/248, 47.2% male participants; *P*<.001) and better recognition of specific AI terms (81/156, 51.9% female vs 75/156, 48.1% male; *P*=.001). However, there was no statistical difference in understanding underlying AI technologies between genders (61.4% female vs 38.6% male; *P*=.50). Male participants demonstrated greater awareness of everyday AI applications like speech recognition and antispam filters (48/86, 55.8% vs 38/86, 44.2% female participants; *P*<.001). First-cycle students reported greater familiarity with technology than those in the second and third cycles (116/248, 46.8% vs 81/248, 32.7% and 51/248, 20.6%, respectively; *P*<.001). Additionally, first-cycle students demonstrated a better understanding of AI terms such as deep learning compared to their peers (83/156, 53.2% vs 51/156, 32.7% and 22/156, 14.1%, respectively; *P*<.001).

**Table 2.** Comparison of artificial intelligence (AI) knowledge among medical students by gender, academic cycle, and familiarity with technology.

Question and response	Gender, n (%)^a^		*P* value	Academic cycle, n (%)^a^			*P* value	Familiarity with technology, n (%)^a^		*P* value
	Female (n=363)	Male (n=217)		First (n=213)	Second (n=228)	Third (n=139)		Knowledgeable (n=332)	Not knowledgeable (n=248)	
Knowledgeable in technology?			*<.001* ^b^				*<.001*	—^c^	—	—
Yes	131 (52.8)	117 (47.2)		116 (46.8)	81 (32.7)	51 (20.6)				
No	232 (69.9)	100 (30.1)		97 (29.2)	147 (44.3)	88 (26.5)				
Familiar with AI terms?			*.001*				*<.001*			*<.001*
Yes	81 (51.9)	75 (48.1)		83 (53.2)	51 (32.7)	22 (14.1)		109 (69.9)	47 (30.1)	
No	282 (66.5)	142 (33.5)		130 (30.7)	177 (41.7)	117 (27.6)		139 (32.8)	285 (67.2)	
Understanding of AI and deep learning?			*<.001*				.15			*<.001*
Great extent	25 (39.1)	39 (60.9)		31 (48.4)	21 (32.8)	12 (18.8)		54 (84.4)	10 (15.6)	
Low extent	177 (66.8)	88 (33.2)		94 (35.5)	99 (37.4)	72 (27.1)		138 (52.1)	127 (47.9)	
Not at all	161 (64.1)	90 (35.9)		88 (35.1)	108 (43)	55 (21.9)		56 (22.3)	195 (77.7)	
Aware of AI in everyday applications?			*<.001*				.39			*<.001*
Great extent	38 (44.2)	48 (55.8)		32 (37.2)	35 (40.7)	19 (22.1)		71 (82.6)	15 (17.4)	
Low extent	217 (66.2)	111 (33.8)		126 (38.4)	118 (36)	84 (25.6)		148 (45.1)	180 (54.9)	
Not at all	108 (65.1)	58 (34.9)		55 (33.1)	75 (45.2)	36 (21.7)		29 (17.5)	137 (82.5)	
Aware of AI in medical research?			.14				.11			*<.001*
Yes	193 (59.9)	129 (40.1)		130 (40.4)	117 (36.3)	75 (23.3)		193 (59.9)	129 (40.1)	
No	170 (65.9)	88 (34.1)		83 (32.2)	111 (43.0)	64 (24.8)		55 (21.3)	203 (78.7)	
Great extent	46 (54.8)	38 (45.2)		28 (33.3)	36 (42.9)	20 (23.8)		56 (66.7)	28 (33.3)	
Aware of AI as a topic of debate in the medical community?			.23				.16			*<.001*
Low extent	191 (62.8)	38 (37.2)		123 (40.5)	105 (34.5)	76 (25.0)		150 (49.3)	154 (50.7)	
Not at all	126 (65.6)	66 (34.4)		62 (32.3)	87 (45.3)	43 (22.4)		42 (21.9)	150 (78.1)	

a Percentages are calculated based on the total number in each row per category.

b Italicized values indicate statistically significant differences between subgroups based on the corresponding *P* values (<.05).

c Not applicable.

**Figure 2.**
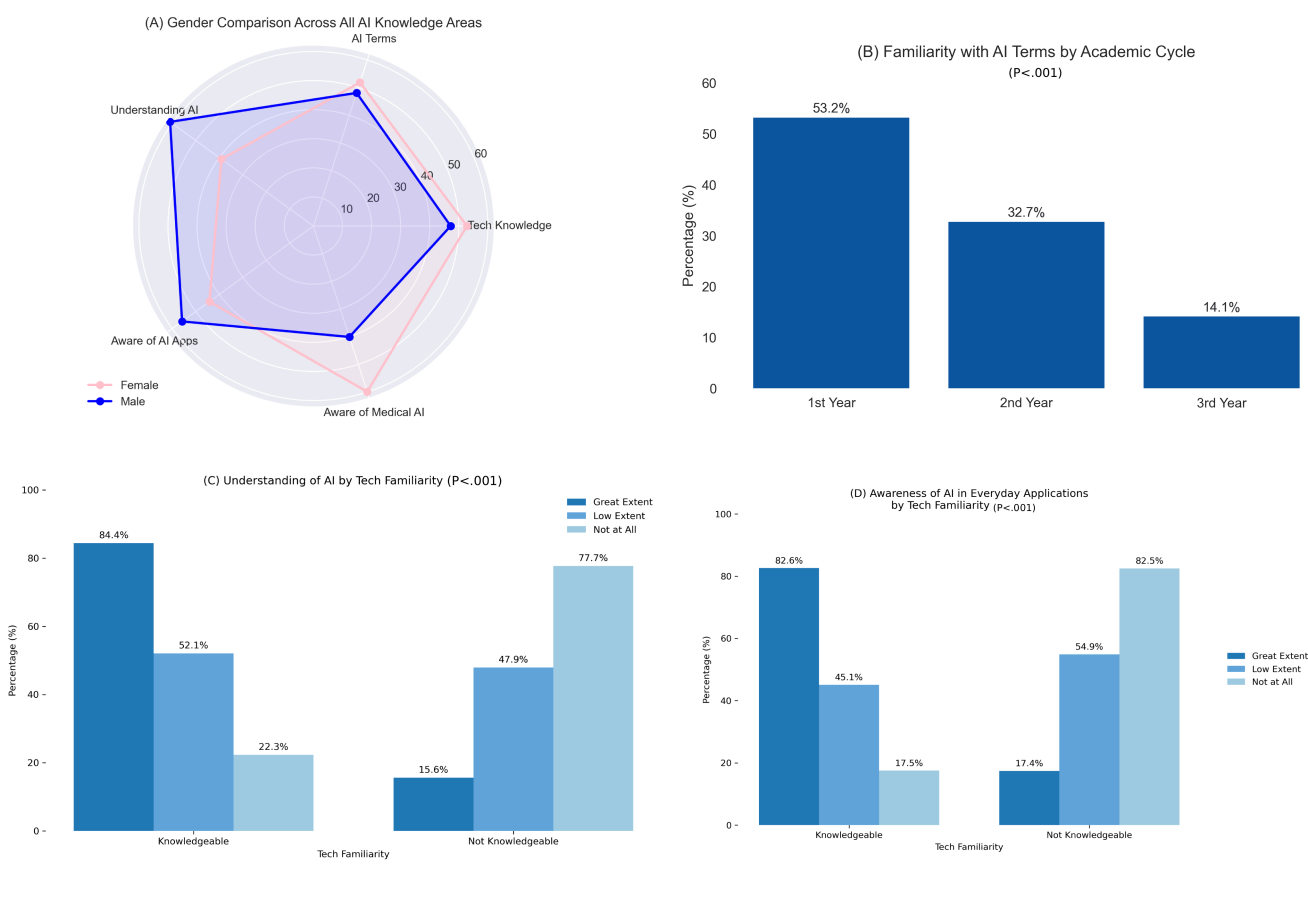
Statistically significant findings in AI knowledge among medical students. (A) Gender differences in technology knowledge, (B) academic cycle impact on AI familiarity, (C) technology familiarity impact on AI understanding, and (D) technology familiarity impact on awareness of everyday AI applications. AI: artificial intelligence.

Figure 2 illustrates the statistically significant findings from Table 2, highlighting key differences in AI knowledge across gender, academic cycle, and technology familiarity.

To objectively assess students’ knowledge of AI concepts, a four-item questionnaire was administered. A substantial majority (n=511, 88%) accurately identified DL as a method for automated pattern recognition essential for biomedical image analysis. Similarly, 88% (n=511) recognized the importance of extensive annotated medical image databases for applying DL in radiology. A notable proportion (n=415, 71.5%) acknowledged the challenges associated with interpreting the decision-making processes of DL models. Additionally, 68% (n=394) understood the limitations of DL in deductive reasoning despite its strengths in pattern recognition. Objective comparison of AI knowledge across demographic variables revealed no significant gender differences. Students in earlier academic cycles (first and second) demonstrated a marginally better comprehension of the complexities underlying DL models compared to their counterparts in the third cycle (*P*=.009). However, this difference was not observed in other AI-related concepts. Regarding technology proficiency, an objective analysis indicated that participants with prior technology exposure were significantly more likely to accurately identify the need for large annotated medical image datasets (*P*=.01). Conversely, those without such experience exhibited a stronger understanding of DL’s limitations in deductive reasoning (*P*=.03).

### Perception of AI Among Medical Students

A majority of students (n=482, 83.1%) viewed AI as a collaborative tool rather than a competitor, while a smaller proportion (n=98, 16.9%) saw it as a potential rival to physicians. When considering the future of noninterventional medicine, 227 (39.1%) anticipated its replacement within a decade, whereas 353 (60.9%) disagreed. Overall, 81% of students believed AI would not supplant human physicians.

However, 488 (84.1%) anticipated AI significantly transforming medicine, while 92 (15.9%) held a different view. Student attitudes toward AI development were divided: 271 (46.7%) expressed concern, while 309 (53.3%) were optimistic. Moreover, 315 (54.3%) were enthusiastic about AI, compared to 265 (45.7%) who were less excited.

Regarding AI’s perceived impact, opinions ranged from negligible (n=22, 3.8%) to substantial (n=207, 35.7%), with most students (n=302, 52.1%) perceiving a moderate influence. When examining these perceptions by gender, a significant majority of both male and female respondents viewed AI as a partner in medicine rather than a competitor (*P*=.94). However, women were more likely to perceive AI as a revolutionary force in medicine (315/488, 64.5%) compared to men (173/488, 35.5%), which was a statistically significant difference (*P*=.02).

While both genders equally anticipated the replacement of noninterventional doctors by AI within a decade (*P*=.99), women expressed greater concern about AI rendering human doctors obsolete (60/111, 54.1%) than men (51/111, 45.9%) a statistically significant difference (*P*=.04). Reactions to AI developments were similar across genders, with no significant differences in fear (*P*=.56) or excitement (*P*=.31).

Perceptions also varied based on technology familiarity. While there was no significant difference in viewing AI as a partner (*P*=.67), students less familiar with technology were more likely to believe AI will revolutionize medicine (260/488, 53.3% vs 228/488, 46.7%; *P*<.001) and make human doctors unnecessary (74/111, 66.7% vs 37/111, 33.3%; *P*=.03). Nonexperts also exhibited greater fear (172/271, 63.5% vs 99/271, 36.5%; *P*=.005) but less enthusiasm (138/315, 43.8% vs 177/315, 56.2%; *P*<.001) for AI developments compared to technology-savvy students. The association of perception of AI with different variables is given in Table 3.

**Table 3.** Comparative analysis of artificial intelligence (AI) perceptions among medical students by gender, familiarity with technology, and academic cycle.

Question and response	Gender, n (%)^a^		*P* value	Familiarity with technology, n (%)^a^		*P* value	Academic cycle, n (%)^a^			*P* value
	Male (n=217)	Female (n=363)		Knowledgeable (n=248)	Not knowledgeable (n=305)		First (n=213)	Second (n=228)	Third (n=139)	
I perceive AI in medicine as a partner rather than a competitor.			.94			.67				.14
Yes	180 (37.3)	302 (62.7)		208 (43.2)	247 (56.8)		172 (35.7)	187 (38.8)	123 (25.5)	
No	37 (37.8)	61 (62.2)		40 (40.8)	58 (59.2)		41 (41.8)	41 (41.8)	16 (16.3)	
AI will revolutionize medicine in general.			*.02^b^*			*<.001*				*.002*
Yes	173 (35.5)	315 (64.5)		228 (46.7)	260 (53.3)		173 (35.5)	185 (37.9)	130 (26.6)	
No	44 (47.8)	48 (52.2)		20 (21.7)	72 (78.3)		40 (43.5)	43 (46.7)	9 (9.8)	
Noninterventional physicians will be replaced within a decade by AI.			.99			.72				*.006*
Yes	85 (37.4)	142 (62.6)		95 (41.9)	132 (58.1)		101 (44.5)	82 (36.1)	44 (19.4)	
No	132 (37.4)	221 (62.6)		153 (43.3)	200 (56.7)		112 (31.7)	146 (41.4)	95 (26.9)	
AI will render human physicians obsolete.			*.04*			*.03*				.16
Yes	51 (45.9)	60 (54.1)		37 (33.3)	74 (66.7)		43 (38.7)	49 (44.1)	19 (17.1)	
No	166 (35.4)	303 (64.6)		211 (45.0)	258 (55.0)		170 (36.2)	179 (38.2)	120 (25.6)	
AI developments frighten me.			.56			*.005*				*<.001*
Yes	98 (36.2)	173 (63.8)		99 (36.5)	172 (63.5)		121 (44.6)	(96 (35.4)	54 (19.9)	
No	119 (38.5)	190 (61.5)		149 (48.2)	160 (51.8)		92 (29.8)	132 (42.7)	85 (27.5)	
AI developments make medicine more exciting for me.			.31			*<.001*				*.005*
Yes	112 (35.6)	203 (64.4)		177 (56.2)	138 (43.8)		111 (35.2)	112 (35.6)	92 (29.2)	
No	105 (39.6)	160 (60.4)		71 (26.8)	194 (73.2)		102 (38.5)	116 (43.8)	47 (17.7)	

a Percentages are calculated based on the total number in each row per category.

b Italicized values indicate statistically significant differences between subgroups based on the corresponding *P* values (<.05).

Notably, women showed a higher perception of AI revolutionizing medicine (315/488, 64.5% vs 173/488, 35.5%; *P*=.02), while technological familiarity significantly impacted perspectives. Students less familiar with technology were more likely to believe AI would revolutionize medicine (260/488, 53.3% vs 228/488, 46.7%; *P*<.001) and potentially render physicians obsolete (74/107, 66.7% vs 33/107, 33.3%; *P*=.03). Academic cycle progression revealed declining enthusiasm, with first-cycle students showing 43.5% (40/92) positive perception, decreasing to 9.8% (9/92) by the third cycle. Technology-naive students demonstrated greater fear of AI developments (172/271, 63.5% vs 99/271, 36.5%; *P*=.005) and less excitement about AI in medicine (138/315, 43.8% vs 177/315, 56.2%; *P*<.001), highlighting the complex relationship between technological exposure and perception.

### AI in Medical Education

In exploring the integration of AI in medical education among Moroccan students, findings revealed strong enthusiasm and perceived benefits. A significant majority (n=524, 90.3%) expressed support for incorporating AI into their curriculum, while an even higher percentage (n=539, 92.9%) believed AI education would positively impact their careers. Regarding its impact on learning conditions, nearly all students (n=546, 94.1%) anticipated AI to enhance their educational experiences. However, when assessing readiness to utilize AI tools upon graduation, opinions were divided: 279 (48.1%) felt capable, whereas 301 (51.9%) did not.

Regarding awareness of AI in medical practice, only 168 (29%) considered themselves well-informed, with the majority (n=412, 71%) feeling less knowledgeable. Examples cited by informed students included AI’s role in medical imaging assistance, robotic surgery, and medical simulation.

Comparative analysis showed that gender did not significantly influence perceptions as responses were similar between men and women across all questions, with *P* values ranging from .15 to .91.

Differences emerged when analyzing responses by academic cycle. While students from all cycles were generally positive about incorporating AI into medical education (*P*=.23), their perceptions varied significantly regarding AI’s impact on medical training (*P*=.04) and its influence on career prospects (*P*=.048). Additionally, there were notable differences in readiness to use AI tools upon graduation (*P*=.03). Specifically, students in the third cycle showed lower confidence (76/279, 27.2%) compared to those in the second cycle (115/279, 41.2%) and first cycle (88/279, 31.5%).

Familiarity with technology also played a role in shaping perceptions. Technology-savvy students were more likely to support the inclusion of AI in the curriculum (*P*=.16), believe in its career benefits (*P*=.26), and expect it to improve learning conditions (*P*=.84). Notably, students familiar with technology felt more prepared to use AI tools upon graduation (*P*<.001), with 56.6% (158/279) of technology-savvy students expressing confidence compared to 29.9% (90/301) of those less familiar with technology. Table 4 presents the results of the comparative analysis.

**Table 4.** Comparative analysis of perception of artificial intelligence (AI) in medical education by gender, academic cycle, and familiarity with technology.

Question and response	By gender, n (%)^a^		*P* value	By academic cycle, n (%)^a^			*P* value	By familiarity with technology, n (%)^a^		*P* value
	Female (n=363)	Male (n=217)		First (n=213)	Second (n=228)	Third (n=139)		Knowledgeable (n=248)	Not knowledgeable (n=332)	
AI should be part of medical education.			.39				.23			.16
Yes	325 (62.0)	199 (38.0)		193 (35.8)	216 (40.1)	130 (24.1)		229 (43.7)	295 (56.3)	
No	38 (67.9)	18 (32.1)		20 (48.8)	12 (29.3)	9 (22.0)		19 (33.9)	37 (66.1)	
AI education will benefit my career.			.91				*.04* ^b^			.26
Yes	337 (62.5)	202 (37.5)		184 (35.1)	210 (40.1)	130 (24.8)		227 (42.1)	312 (57.9)	
No	26 (63.4)	15 (36.6)		29 (51.8)	18 (32.1)	9 (16.1)		21 (51.2)	20 (48.8)	
AI will improve learning conditions.			.64				*.048*			.85
Yes	343 (62.8)	203 (37.2)		194 (35.5)	220 (40.3)	132 (24.2)		234 (42.9)	312 (57.1)	
No	20 (58.8)	14 (41.2)		19 (55.9)	8 (23.5)	7 (22.6)		14 (41.2)	20 (58.8)	
I will be able to use AI tools in health care after graduation.			.15				*.03*			*<.001*
Yes	183 (65.6)	96 (34.4)		88 (31.5)	115 (41.2)	76 (27.2)		158 (56.6)	121 (43.4)	
No	180 (59.8)	121 (40.2)		125 (41.5)	113 (37.5)	63 (20.9)		90 (29.9)	211 (70.1)	

a Percentages are calculated based on the total number in each row per category.

b Italicized values in the table indicate statistically significant differences between subgroups based on the corresponding *P* values (<.05).

Table 4 reveals the evolution of medical students’ perceptions about AI in medical education across academic cycles and technological familiarity. There was a declining belief that AI education would benefit careers, decreasing from 35.1% (184/524) in the first cycle to 24.8% (130/524) in the third cycle (*P*=.04). Similarly, there was a reduction in perceiving AI’s potential to improve learning conditions, from 35.5% (194/546) in the first cycle to 24.2% (132/546) in the third cycle (*P*=.048). A progressive decrease was also noted in confidence about using AI tools post graduation, dropping from 31.5% (88/279) in the first cycle to 27.2% (76/279) in the third cycle (*P*=.03). There was also a significant impact of technological familiarity on confidence in using AI tools after graduation, with knowledgeable students showing 56.6% (158/279) confidence compared to 43.4% (121/279) among those less familiar (*P*<.001).

### The Influence of AI on Students’ Perceptions and Specialty Choices

The impact of AI on students’ perceptions and specialty choices in medical education reveals several noteworthy findings. First, radiology was overwhelmingly viewed as the specialty most likely to be affected by AI, with 63% (n=365) of students selecting it as the top choice, followed by surgery at 17% (n=99), and anatomical pathology at 11% (n=62). Concerning the replacement of medical specialties by AI, 57% (n=331) of students believed certain specialties would be substituted, while 43% (249) held differing views. Regarding the influence of AI advancements on specialty selection, a significant majority (n=327, 56%) indicated they were not inclined to choose specialties heavily influenced by AI progress, contrasting with 43% (n=249) who felt otherwise.

Analysis by gender showed no substantial influence on specialty choices. Similarly, technological familiarity did not significantly impact the perception of AI replacing medical specialties (*P*=.35). However, significant differences emerged when considering whether students would avoid AI-affected specialties, with 48% (119/327) of technology-savvy students versus 62.7% (208/327) of nonsavvy students (*P*<.001) indicating potential avoidance. Additionally, while there was no significant difference among different academic cycles regarding the replacement of medical specialties by AI (*P*=.70), a statistically significant difference was observed (*P*<.001) concerning the avoidance of AI-impacted specialties across academic stages. Table 5 presents the results of the comparative analysis.

**Table 5.** Influence of artificial intelligence (AI) on students’ perceptions and specialty choices by gender, technology familiarity, and academic cycle.

Question and response	By gender, n (%)^a^		*P* value	By academic cycle, n (%)^a^			*P* value	By familiarity with technology, n (%)^a^		*P* value
	Female (n=363)	Male (n=217)		First (n=213)	Second (n=228)	Third (n=139)		Knowledgeable (n=248)	Not knowledgeable (n=332)	
Certain specialties will be replaced by AI.			.16				.23			.35
Yes	199 (60.1)	132 (39.9)		126 (38.1)	129 (39.0)	76 (23.0)		136 (41.1)	195 (58.9)	
No	164 (65.9)	85 (34.1)		87 (34.9)	99 (39.8)	63 (25.3)		112 (45.0)	137 (55.0)	
I might not choose a specialty like radiology because of AI.			.82				*<.001* ^b^			*<.001*
Yes	206 (63.0)	121 (37.0)		138 (42.2)	128 (39.1)	61 (18.7)		119 (48.0)	208 (62.7)	
No	157 (62.1)	96 (37.9)		75 (29.6)	100 (39.5)	78 (30.8)		129 (52.0)	124 (37.3)	

a Percentages are calculated based on the total number in each row per category.

b Italicized values indicate statistically significant differences between subgroups based on the corresponding *P* values (<.05).

## Discussion

This study aimed to assess the knowledge and perceptions of AI among medical students in a medical school in Morocco. The findings reveal a generally low level of AI knowledge, with significant variations based on gender and year of study, yet students maintain a positive perception of AI, particularly regarding its potential to revolutionize medicine and its integration into medical education.

The study identified a female majority among the participants, comprising 63% of the sample. This gender distribution aligns with national data from the Haut-Commissariat au Plan, which reported a 67.8% female presence in medical faculties [23]. Similar trends are observed internationally, as evidenced by studies conducted by Bisdas et al [24] (66.5%), Pinto Dos Santos et al [17] (63%), and Buabbas et al [25] (88.9%). The consistent female predominance across different contexts suggests a broader trend in the medical education landscape.

Our study shows that although a large majority of students (96%) have been exposed to AI-related terms, there is a significant gap in understanding its core concepts. Specifically, 73% of students are unfamiliar with terms like machine learning and neural networks. This finding aligns with a study in Syria, where 70% of participants had a basic understanding of AI, but only 34.7% knew about ML and DL, and just 23.7% understood AI applications [26]. An additional study from India reported that 73.6% of respondents felt unknowledgeable about AI’s basic principles and applications, with 81.1% lacking understanding of AI’s limitations [27]. Similarly, a German study found that while 83.3% of students understood the term “artificial intelligence,” familiarity with more specific terms was lower: 65.9% knew about “machine learning,” 42.3% about “neural networks,” and only 18.7% about “deep learning” [28]. These findings suggest that despite widespread awareness of AI, students’ knowledge is often superficial, likely influenced more by popular media than by academic sources.

The gender differences in AI knowledge observed in our study, where female participants reported higher familiarity with technology and AI terms but male participants were more aware of AI applications, reflect patterns seen in other research. For instance, a UK study found that male students were more aware of AI applications despite both genders having similar understanding of AI principles [19]. This aligns with our study, suggesting that while women may be more comfortable with technology and AI terminology, men may have a greater exposure to AI applications in their daily lives. Additionally, research in Germany showed that male students had higher AI knowledge than female students [17]. This discrepancy between self-reported familiarity and actual knowledge underscores the need to distinguish between perceived and objective understanding of AI concepts, as both genders may have similar objective knowledge but different experiences and exposures.

Despite limited knowledge, students had a generally positive perception of AI. Most of them (83%) saw AI as a partner rather than a competitor, which is consistent with the 72.2% reported in the Bisdas et al [24] study. Additionally, 84% believed AI would revolutionize medicine, similar to findings from Bisdas et al [24] (83.9%) and Buabbas et al [25] (99.1%). However, 61% thought AI might replace noninterventionist physicians, a concern more prevalent in Morocco compared to other countries (eg, 32.5% in Bisdas et al’s [24] study and 6.5% in Pinto Dos Santos et al’s [17] study). This reflects a nuanced understanding among students, recognizing AI’s potential and its limitations in clinical practice.

Our study also found that 56% of students would avoid specialties heavily influenced by AI, with this sentiment more pronounced among those less familiar with technology (62.7%) compared to technology-savvy students (48%). This avoidance behavior has been documented in other studies as well. For example, a survey of radiology students in Canada found that 45% were concerned about AI replacing their future roles, leading to hesitation in choosing radiology as a specialty [29].

Enthusiasm for AI in medical education was high, with 90% of students supporting its inclusion, exceeding the 75% reported among UK medical students and 69.4% among Malaysian students [19,30]. However, only 48% felt ready-to-use AI tools upon graduation, with confidence declining to 27.2% in the third cycle. This aligns with findings from Malaysia, where 44.5% of students felt prepared to apply AI in practice, and a Qatari study, where 43.8% stated they would consistently use AI in decision-making [30,31].

This study highlights key implications for medical education in Morocco. The significant knowledge gap in AI among students signals the need for urgent curricular reform, particularly by introducing AI-specific modules early in medical training. The positive perception of AI as a partner suggests that future doctors are ready to embrace AI tools in clinical practice, potentially enhancing patient care. Additionally, comparing these findings with global studies underscores the importance of aligning Moroccan medical education with international standards.

However, this study has limitations. The use of snowball sampling may have introduced selection bias, affecting the generalizability of the results. Also, focusing on a specific region might not fully represent the broader context of medical education in Morocco. Finally, reliance on self-reported data could lead to inaccuracies as students might over- or underestimate their knowledge and perceptions of AI.

In conclusion, this study sheds light on the current state of AI knowledge and perceptions among medical students in Morocco, revealing both challenges and opportunities. While there is a general enthusiasm for AI, the significant knowledge gap underscores the need for curricular reform and targeted educational interventions. The positive perceptions of AI suggest that students are ready to engage with these technologies, paving the way for a future where AI plays a central role in medical practice.

## Abbreviations

AI: artificial intelligence
DL: deep learning
FMPA: Faculty of Medicine and Pharmacy, Agadir
ML: machine learning
